# Synthetic microbial consortia derived from rhizosphere soil protect wheat against a soilborne fungal pathogen

**DOI:** 10.3389/fmicb.2022.908981

**Published:** 2022-08-31

**Authors:** Chuntao Yin, Christina H. Hagerty, Timothy C. Paulitz

**Affiliations:** ^1^North Central Agriculture Research Laboratory, USDA-ARS, Brookings, SD, United States; ^2^Columbia Basin Agricultural Research Center, Oregon State University, Adams, OR, United States; ^3^Wheat Health, Genetics and Quality Research Unit, USDA-ARS, Pullman, WA, United States

**Keywords:** synthetic microbial communities, microbes, *Rhizoctonia solani*, volatiles, cell-free supernatants

## Abstract

Synthetic microbial communities (SynComs) could potentially enhance some functions of the plant microbiome and emerge as a promising inoculant for improving crop performance. Here, we characterized a collection of bacteria, previously isolated from the wheat rhizosphere, for their antifungal activity against soilborne fungal pathogens. Ten SynComs with different compositions from 14 bacterial strains were created. Seven SynComs protected wheat from *Rhizoctonia solani* AG8 infection, although SynComs were not more effective than single strains in reducing wheat root rot disease. Further, the mechanisms of interaction of the tested bacteria with each other and plants were explored. We found that nine bacteria and nine SynComs impacted the root growth of *Arabidopsis*. Nine bacteria and four SynComs significantly inhibited the growth of AG8 by producing volatiles. The cell-free supernatants from six bacteria inhibited the growth of AG8. Together, this study provided the potential for improving crop resilience by creating SynComs.

## Introduction

Rhizoctonia bare patch and root rot disease, caused by *Rhizoctonia solani* AG8, is a major yield limitation of wheat production in direct-seeded or minimal tillage farming systems (Murray and Brennan, 2010; Paulitz et al., 2010). Genetic resistance to AG8 in wheat cultivars is currently absent for growers (Okubara and Jones, 2011). There are very few control methods to prevent this disease. Tillage is considered an effective method to reduce disease by disrupting fungal hyphal networks (Schroeder and Paulitz, 2008; Almasudy et al., 2015). The adoption of minimum or no-till seeding practices for improving soil quality and reducing soil erosion and labor costs increases soilborne disease pressure in cropping systems (Pittelkow et al., 2015b). Fungicidal seed treatments can improve seedling health, but do not mitigate bare patch in late growth stages (Paulitz and Scott, 2006). Suppression of Rhizoctonia bare patch in wheat fields under continuous wheat cropping in Australia (Roget, 1995) and the Pacific Northwest (PNW) in the United States (Schillinger and Paulitz, 2014) has been reported. Soil microbial communities have been involved in the development of Rhizoctonia suppressive soils (Yin et al., 2013, 2021; Donn et al., 2014; Hayden et al., 2018) and some bacterial species were found to reduce root rot disease. The interaction of three bacteria, *Pantoea*, *Exiguobacterium*, and Microbacteria, contributed to wheat root rot disease suppression (Barnett et al., 2006). Our previous studies characterized a group of bacteria from plant rhizospheres that possess antifungal activities (Yin et al., 2013, 2021). Most recently, Zhang et al. (2021) found that *Bacillus subtilis* strain NCD-2 and *Pseudomonas protegens* strain FD6 significantly inhibited the growth of AG8 *in vitro* and decreased the abundance of AG8 in the rhizosphere. These findings support development of an alternative to managing soilborne root diseases and raise interest in harnessing soil microbes to improve crop health, resiliency, and productivity.

Beneficial soil microbes, such as plant growth-promoting (PGP) microbes and arbuscular mycorrhizal fungi (AMF), are widely studied and can enhance plant growth and increase crop yields (Lugtenberg and Kamilova, 2009; Hayat et al., 2010; de Souza et al., 2015; Hyder et al., 2020). PGP microbes promote plant growth through a wide variety of mechanisms (Glick, 2012; Alemneh et al., 2020). The mechanisms include facilitating nutrition (e.g., nitrogen fixation, phosphate solubilization, and siderophores; Rodrıguez and Fraga, 1999; Glick, 2012; Maindad et al., 2014) and modulating phytohormone levels (e.g., indole acetic acid, cytokinins, gibberellins, and ethylene; Persello-Cartieaux et al., 2003; Glick, 2012). Additionally, PGP microbes can stimulate plant growth and protect the plant against phytopathogens by producing antibiotics (Haas and Keel, 2003; Mazurier et al., 2009), hydrogen cyanide (HCN; Abd El-Rahman et al., 2019), and lytic enzymes (Glick, 2012) or by inducing systemic resistance in plants (Pieterse et al., 2014). These properties regulated by PGP microbes are effective at controlling diseases caused by different phytopathogens (Bakker et al., 2007). To date, many PGP microbes have been isolated and some of them have been commercialized as biofertilizers, biostimulants, and biocontrol agents, such as *Azospirillum* spp., *Bacillus* spp., *Burkholderia* spp., *Streptomyces* spp., and *Pseudomonas* spp. (Glick, 2012; Bonanomi et al., 2018). However, the practical application of PGP microbes in fields is still limited because the results are inconsistent, for example as the added microbes can be excluded by the more-resilient existing microbiome over time (Kaminsky et al., 2019; Ke et al., 2021).

To alleviate the limitations, synthetic microbial communities (SynComs), small consortia of microorganisms, are increasingly recognized as a promising way to preserve some specific functions of the plant microbiome but reduce the complexity of the microbial communities (Großkopf and Soyer, 2014; de Souza et al., 2020). This strategy allows selection of specific microorganisms in the laboratory according to their abilities to promote plant growth, protect plants from pathogen infection, or provide nutrition for plants. Then, researchers consolidate diverse traits of microbes in SynComs. Several studies have successfully shown that designed SynComs improve plant performance, such as growth promotion and disease resistance. For example, Tsolakidou et al. (2019) designed two SynComs with different bacterial genera that promoted tomato growth or suppressed Fusarium wilt symptoms of tomato. Similarly, positive effects of microbial consortia were reported in other studies (Castrillo et al., 2017; Kong et al., 2018; Carrión et al., 2019; Thomloudi et al., 2019). More recently, Carrión et al. (2019) reported that the consortium of *Flavobacterium* and *Chitinophaga* consistently protected sugar beet from infection by the fungus *Rhizoctonia solani*. Senghor et al. (2020) demonstrated that a biocontrol product consisting of a mixture of four atoxigenic *Aspergillus flavus* strains reduced aflatoxin contamination in groundnut and maize. Hori et al. (2021) reported that the fungal pairs, the genera *Fusarium* and *Curvularia*, showed a greater effect on plants than the single strains. Additionally, emergent community properties, in which the functions of microbial communities go beyond mere additivity of individuals, can be achieved through the interaction of several species (Madsen et al., 2016; Baveye et al., 2018). Fujiwara et al. (2016) demonstrated that interactions of non-antagonistic bacteria resulted in antagonism against pathogens. Similarly, Yang et al. (2021) found that a four-species bacterial consortium (*Stenotrophomonas rhizophila*, *Xanthomonas retroflexus*, *Microbacterium oxydans*, and *Paenibacillus amylolyticus*) induced drought tolerance of *Arabidopsis*, whereas the individuals did not protect plants from drought stress. These emergent properties are likely the key to improving the success of PGP microbes in field applications. However, the inoculum persistence, spread, and displacement of resident microbes by competitive exclusion should be considered. Interest in designing and applying SynComs for agricultural sustainability is emerging, but there is a need to assess microbial impacts on plants, the practical perspective of microbial consortia applied effectively in the agricultural ecosystems, and the molecular and chemical basis underpinning the interactions and stability of microbial consortia.

In this study, we characterized a collection of bacteria, previously isolated from the wheat rhizosphere, for antifungal activity against soil-borne fungal pathogens (Yin et al., 2013, 2021). The protection of wheat from *Rhizoctonia solani* AG8 infection using 14 bacteria was evaluated. Subsequently, 10 SynComs with different compositions from 14 bacterial isolates were created. We hypothesized that the designed SynComs can increase the protection of wheat against AG8 infection. The aims of this study are: (1) to evaluate if bacteria isolated from the wheat rhizosphere protect a susceptible wheat cultivar against AG8; (2) to test if the created SynComs enhance disease protection; and (3) to explore the basis of bacterial antifungal activities. This study explored the potential for developing microbiome technologies to improve plant resiliency against adverse environmental stress leading to sustainable agriculture.

## Materials and methods

### Plant, fungal, and bacterial strains

Wheat cultivar Alpowa, susceptible to soilborne fungal pathogen *R. solani* AG8, was used in this study. All wheat seeds were derived from the same seed source to reduce plant variation. Seeds were treated with 5% sodium hypochlorite for 3 min for surface disinfestation and rinsed three times with sterilized double-distilled water (ddH_2_O) before germination. All wheat plants were grown in a greenhouse in 16/8 h light/darkness at 16°C. *Arabidopsis thaliana* Col-0 accession (hereafter *Arabidopsis*) was used to evaluate the influence of bacteria on root growth. The soil used in this study was collected from the Washington State University Dryland Research Station near Lind (47°0’N, 118°34’W), WA, United States, as described previously (Yin et al., 2021). The fungal pathogen *R. solani* AG8 was used and grown in potato dextrose agar medium (PDA, Sigma-Aldrich, St. Louise, MO) at 25°C. The inoculum of *R. solani* AG8 was prepared with twice-autoclaved millet seeds, as described previously (Yin et al., 2021). The bacterial strains isolated from the wheat rhizosphere (Yin et al., 2013, 2021) were grown on 1/4 x tryptic soy agar/1 x tryptic soy broth (TSA/TSB, BD, Franklin Lakes, NJ) at 25°C.

### Synthetic microbial communities

Ten synthetic communities (SynComs) of bacteria were designed using 14 bacterial strains previously isolated from the wheat rhizosphere (Yin et al., 2013, 2021) based on the following criteria: (1) different strains of the same genus (SynComs 1 and 2; hereafter C1 and C2); (2) relatively strong antifungal activities in single strain test (SynComs 3, 4, 5, 8, and 9; C3, C4, C5, C8, and C9); (3) random combinations (SynComs 6 and 7; C6 and C7); and (4) the combination of all 14 bacterial isolates (SynCom 10; C10). In most cases, the SynComs consist of four strains to reduce the complexity of bacterial interaction and the cost for future applications. The details of 10 SynComs in this study are listed in Table 1. Each bacterium was grown in a Petri dish with 1/4 x TSA medium at 25°C for 48 h. Bacterial cultures were collected in sterile ddH_2_O and centrifuged at 5,000 g for 5 min. Then, bacterial cells were resuspended in sterile ddH_2_O. The optical density OD_600_ of each suspension was adjusted to 0.1 or 1.0 for different treatments. SynComs were obtained by mixing the individuals in equal ratios. Then, the same volume of bacterial suspension was applied for sample treatments.

**Table 1:** The designed synthetic microbial communities (SynComs) in this study.

SynComs ID	Bacterial components
SynCom 1(C1)	*Pseudomonas* sp. B5, *Pseudomonas* sp. B11, *Pseudomonas* sp. B12, *Pseudomonas* sp. P25
SynCom 2(C2)	*Chryseobacterium* sp. B7, *Chryseobacterium soldanellicola* P38, *Chryseobacterium* sp. P43
SynCom 3(C3)	*Sphingomonas* sp. B17, *Cupriavidus campinensis* B20, *Asticcacaulis* sp. B27, *Rhodococcus erythropolis* B43
SynCom 4(C4)	*Cupriavidus campinensis* B20, *Asticcacaulis* sp. B27, *Rhodococcus erythropolis* B43, *Chryseobacterium soldanellicola* P38
SynCom 5(C5)	*Cupriavidus campinensis* B20, *Rhodococcus erythropolis* B43, *Janthinobacterium lividum* BJ, *Chryseobacterium soldanellicola* P38
SynCom 6(C6)	*Streptomyces* sp. B6, *Chryseobacterium* sp. B7, *Pseudomonas* sp. B12, *Sphingomonas* sp. B17
SynCom 7(C7)	*Pseudomonas* sp. B5, *Streptomyces* sp. B6, *Chryseobacterium* sp. B7, *Pseudomonas* sp. B11
SynCom 8(C8)	*Pseudomonas* sp. B12, *Sphingomonas* sp. B17, *Cupriavidus campinensis* B20, *Asticcacaulis* sp. B27
SynCom 9(C9)	*Pseudomonas* sp. B12, *Rhodococcus erythropolis* B43, *Janthinobacterium lividum* BJ, *Pedobacter* sp. P44
SynCom 10(C10)	All 14 bacterial strains

### *In vitro* antagonistic activities of bacteria against soilborne pathogen

The antagonistic activities of bacterial strains against *R. solani* AG8 were tested by *in vitro* dual-culture assay on 1/2 x PDA medium. A 5-mm mycelial plug from 7-day-old fungal culture was placed in the center of the Petri dish. Four drops of 1 μl of bacterial culture (OD_600_ = 0.1) were added at ~5 mm from the rim of the Petri dish in four even directions. For negative controls, Petri dishes were inoculated only with a mycelial plug or with a mycelial plug and sterile ddH_2_O. Paired culture Petri dishes were placed in the dark and incubated at 25°C until the PDA medium for the controls was completely covered with *R. solani* AG8 mycelia. The co-culture Petri dishes were scanned with Epson Perfection V700 Photo scanner (Epson America, Inc., Los Alamitos, CA). The radial growth of the fungal pathogen was measured with a ruler. The inhibitory activity of bacteria was calculated with the equation: 100 × [(R1−R2)/R1], where R1 was the radial growth of pathogen in the control and R2 was the radial growth of pathogen in the dual culture with antagonist. The experiment was repeated twice with three replicates of each treatment.

### Greenhouse suppression assays

The field soil was amended with ground millet inoculum of *R. solani* AG8 to a final concentration of 100 propagules per gram (ppg) of soil. Plastic cones (25 mm diameter and 165 mm long) were filled with a 65-mm-thick column of sterile vermiculite followed by 10 g of *Rhizoctonia*-inoculated soil. The bacteria were scraped from 1/4 × TSA Petri dishes, suspended in ddH_2_O, and centrifuged for 5 min at 5,000 rpm. The pellet was resuspended in sterile ddH_2_O and adjusted to the optical density OD_600_ value of 1.0. Three-day-old pre-germinated wheat seeds were incubated in the bacterial slurries (single strain or SynComs, OD600 = 1.0) for 30 min at 25°C, while the control seeds were treated with an equal amount of sterile water before planting. Wheat seeds were treated with the bacterial slurries or sterile ddH_2_O and soil samples amended with ground autoclaved millet without *Rhizoctonia* served as controls. Three treated wheat seeds were sown in each cone and covered with a 15 mm-thick topping of vermiculite. Each cone received 10 ml of water. Cones were arranged in a randomized complete block design in plastic racks and incubated in a greenhouse with 16/8 h light/darkness at 16°C. Each cone received 10 ml of water twice a week and diluted (1:3 [vol vol^−1^]) Hoagland’s solution once a week. After 3 weeks, the seedlings were removed from the cones, the roots were washed free of soil, and the plants were evaluated for Rhizoctonia root rot disease on a scale of 0 to 8 as described previously (Yin et al., 2013). The fresh root weight from three plants was measured after removed extra water with a paper towel. Each treatment had 10 replicates, and the experiment was conducted twice.

### Indole acetic acid quantification assays

Bacteria were cultured in TSB medium in a shaking incubator (250 rpm) at 25°C for 24 h. Bacterial cultures (1 ml) were centrifuged at 13,000 g for 5 min, and 50 μl of supernatant per sample was added to a 96-well plate followed by 100 μl of Salkowski reagent (1 ml of 0.5 M ferric chloride and 50 ml of 35% perchloric acid) and then incubated at room temperature for 30 min. Absorbance was measured at 530 nm in a Tecan Safire 2 microplate reader (Tecan, Männedorf, Switzerland). The concentration of IAA was determined for each sample after comparison with a standard curve for IAA concentration ranging between 5 and 100 μg ml^−1^.

### *Arabidopsis* growth assays

*Arabidopsis* seeds were surface sterilized and sown on 1/2 x Murashige and Skoog (MS) medium (PhytoTech Labs Inc., Lenexa, KS) containing 0.8% (wt vol^−1^) phytablend (Caisson Laboratories Inc., Rexburg, ID) and 1.5% (wt vol^−1^) sucrose in square Petri dishes (120 × 120 × 17 mm). After 4 days of stratification at 4°C, Petri dishes were transferred and positioned vertically in a growth chamber with 16/8 h light/darkness and light intensity 30 μmol m^−2^ s^−1^ at 25°C. Uniform six-day-old *Arabidopsis* seedlings were transferred to new square Petri dishes containing 1/2 × MS medium. The bacteria were scraped from 1/4 × TSA Petri dishes, suspended in sterile ddH_2_O, and centrifuged for 5 min at 5,000 rpm. The pellet was resuspended in sterile ddH_2_O and adjusted to the optical density OD_600_ value of 0.1. SynComs were prepared by mixing the individuals in equal ratios. Two μl of bacterial suspensions of single strain or SynComs were spotted just below the root tip of each *Arabidopsis* plant. After a week of co-cultivation, the growth of *Arabidopsis* in the Petri dishes was scanned with Epson Perfection V700 Photo scanner. The primary root length was measured using ImageJ software (version 1.53e; Schneider et al., 2012).

### Effects of bacterial volatiles on AG8 growth

The effects of bacterial volatiles on the growth of AG8 were studied using a large Petri dish (150 mm diameter) containing three small Petri dishes (60 mm diameter). Two small Petri dishes with 1/4 × TSA were used as a volatile-producing compartment to culture bacteria. One small Petri dish with 1/2 × PDA was used as a volatile-receiving compartment to grow AG8. The bacteria were scraped from 1/4 × TSA Petri dishes, suspended in sterile ddH_2_O, and centrifuged for 5 min at 5,000 rpm. The pellet was resuspended in sterile ddH_2_O and adjusted to the optical density OD_600_ value of 0.1. SynComs were prepared by mixing the individuals in equal ratios and the final OD_600_ was 0.1. Five drops of 1 μl of bacterial culture were added at the center and ~ 5 mm from the rim of two small Petri dishes in four even directions. Then, the two Petri dishes inoculated with bacteria were placed inside of a large Petri dish and bacteria were grown at 25°C. After 5 days, the third small Petri dish inoculated with a 5-mm AG8 mycelial plug was also placed inside of the large Petri dish and the large Petri dishes were sealed with parafilm. For negative controls, the first two small Petri dishes were inoculated only with 1 μl of sterile ddH_2_O. After a week of co-cultivation, the large Petri dishes were scanned with Epson Perfection V700 Photo scanner. The radial growth of AG8 was measured using ImageJ software. The inhibitory activity of volatiles produced by bacteria was calculated as described above. Each treatment had three replicates. The experiment was repeated twice.

### Antifungal activity of cell-free supernatants

Bacteria were inoculated into TSB medium in 10-ml culture tubes with final OD_600_ = 0.1 and grown in a shaking incubator (250 rpm) at 25°C for 3 days. One ml of culture was centrifuged at 13,000 g for 10 min. The supernatants were immediately filtered through 0.22-μm filters. Then, two 5-mm agar plugs at ~5 mm from the Petri dishes rim were removed using a sterile cork borer (diameter of 8.5 mm) and 100 μl of supernatants were added into each agar well. 100 μl of TSB were added as negative controls. Five-mm mycelial AG8 plugs were placed in the center of the Petri dish. After a week of incubation at 25°C, the Petri dishes were scanned with Epson Perfection V700 Photo scanner and the radial growth of AG8 was measured using ImageJ software. Each treatment had three replicates. The experiment was repeated twice.

### Hydrogen cyanide measurement

Hydrocyanic acid (HCN) produced by bacteria was examined according to the method described by Abd El-Rahman and Shaheen (2016). Briefly, bacteria were scraped from 1/4 × TSA Petri dishes, suspended in sterile ddH_2_O, and centrifuged for 5 min at 5,000 rpm. The pellet was resuspended in sterile ddH_2_O and adjusted to the OD_600_ value of 0.1. Then, bacterial cultures (100 μl) were spread on TSA medium amended with 4.4 g l^−1^ glycine in Petri dishes. Filter papers soaked in the picric acid solution (0.5% picric acid in 2% sodium carbonate) were put into the lid of each Petri dish. These Petri dishes were sealed with parafilm and incubated for 7 days at 25°C. Then, filter papers were observed for color changes from yellow (control) to light brown, brown, or reddish brown which were recorded as weak, moderate, or strong reaction, respectively. Non-bacteria inoculated Petri dishes were used as control. Three replicates were used for each tested bacterium.

### Statistical analysis

Data analysis was performed in R (v4.1.2). Before statistical analysis, Levene’s test was performed for the heteroscedasticity of data with “car” package. The primary root length of Arabidopsis was transformed using a square root transformation. Multiple comparisons for wheat root rot score, fresh root weight, and square-root-transformed primary root length of Arabidopsis were analyzed by one-way ANOVA (analysis of variance), followed by a *post-hoc* Tukey’s HSD test (*p* < 0.05) with “agricolae” and “foreign” packages. The difference in inhibition of AG8 growth treated with bacteria was tested with the Kruskal–Wallis test, followed by a *post-hoc* Dunn test (*p* < 0.05) with “FSA” package. Data were presented as mean ± standard deviation (SD, *n* = 3–10). Correlation analysis was conducted to evaluate relationships between primary root length of Arabidopsis and IAA produced by bacteria using the “cor.test” function with “ggpubr” package.

## Results

### Antagonism assay against AG8 *in vitro*

**Figure 1:**
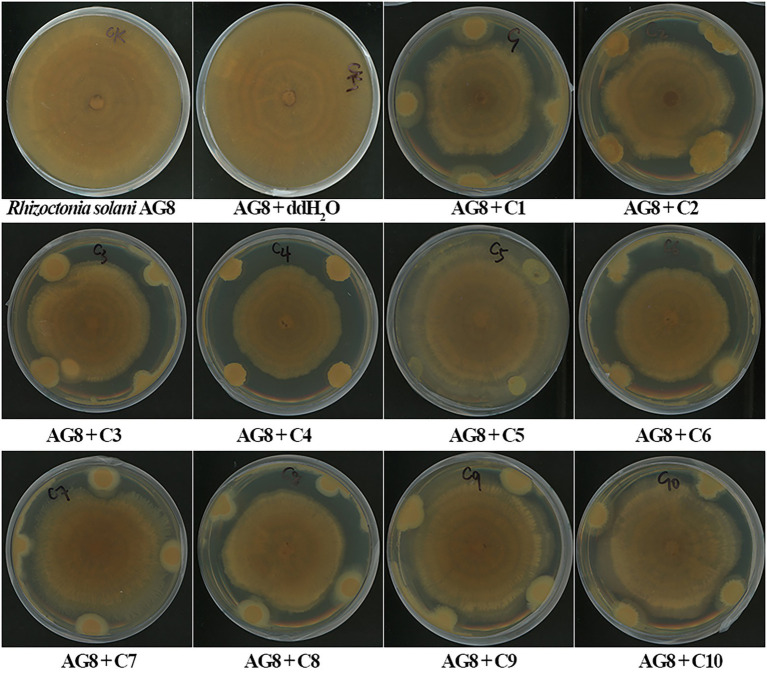
The inhibition of synthetic microbial communities (SynComs) on the growth of *Rhizoctonia solani* AG8 in dual-culture assay. C1: SynCom 1; C2: SynCom 2; C3: SynCom 3; C4: SynCom 4; C5: SynCom 5; C6: SynCom 6; C7: SynCom 7; C8: SynCom 8; C9: SynCom 9; C10: SynCom 10.

*In vitro* dual-culture assay showed eight SynComs, including C1-C4, C6-C8, and C10, inhibited the growth of *R. solani* AG8 to varying degrees (Figure 1). The AG8 growth inhibition ranged from 23.76 ± 12.68 to 37.00% ± 6.57% (Table 2) over the control of fungi only. Maximum fungal mycelial growth inhibition was observed by the treatment of C1 (37.00% ± 6.57%) and C8 (35.01% ± 9.18%), while C5 and C9 did not significantly inhibit AG8 growth. However, *in vitro* assays may not capture the complexity of the interaction between microbial communities and plants.

**Table 2:** Inhibition of SynComs on the radial growth of *R. solani* in dual-culture assays.

Bacteria	% Inhibition of radial growth^a^
Control (*R. solani* AG8 only)	0
Control (ddH_2_O)	0
SynCom 1(C1)	37.00 ± 6.57^*^
SynCom 2(C2)	31.03 ± 13.32^*^
SynCom 3(C3)	24.72 ± 3.88^*^
SynCom 4(C4)	28.79 ± 7.01^*^
SynCom 5(C5)	13.44 ± 2.13
SynCom 6(C6)	34.48 ± 5.91^*^
SynCom 7(C7)	25.15 ± 6.80^*^
SynCom 8(C8)	35.01 ± 9.18^*^
SynCom 9(C9)	17.96 ± 1.25
SynCom 10(C10)	23.76 ± 12.68^*^

a The values are means ± standard deviation (SD, n = 3).

* Indicate significant differences (p ≤ 0.05, Dunn test).

### Inhibitory effect of bacteria on *Rhizoctonia solani* AG8 in soil

**Figure 2:**
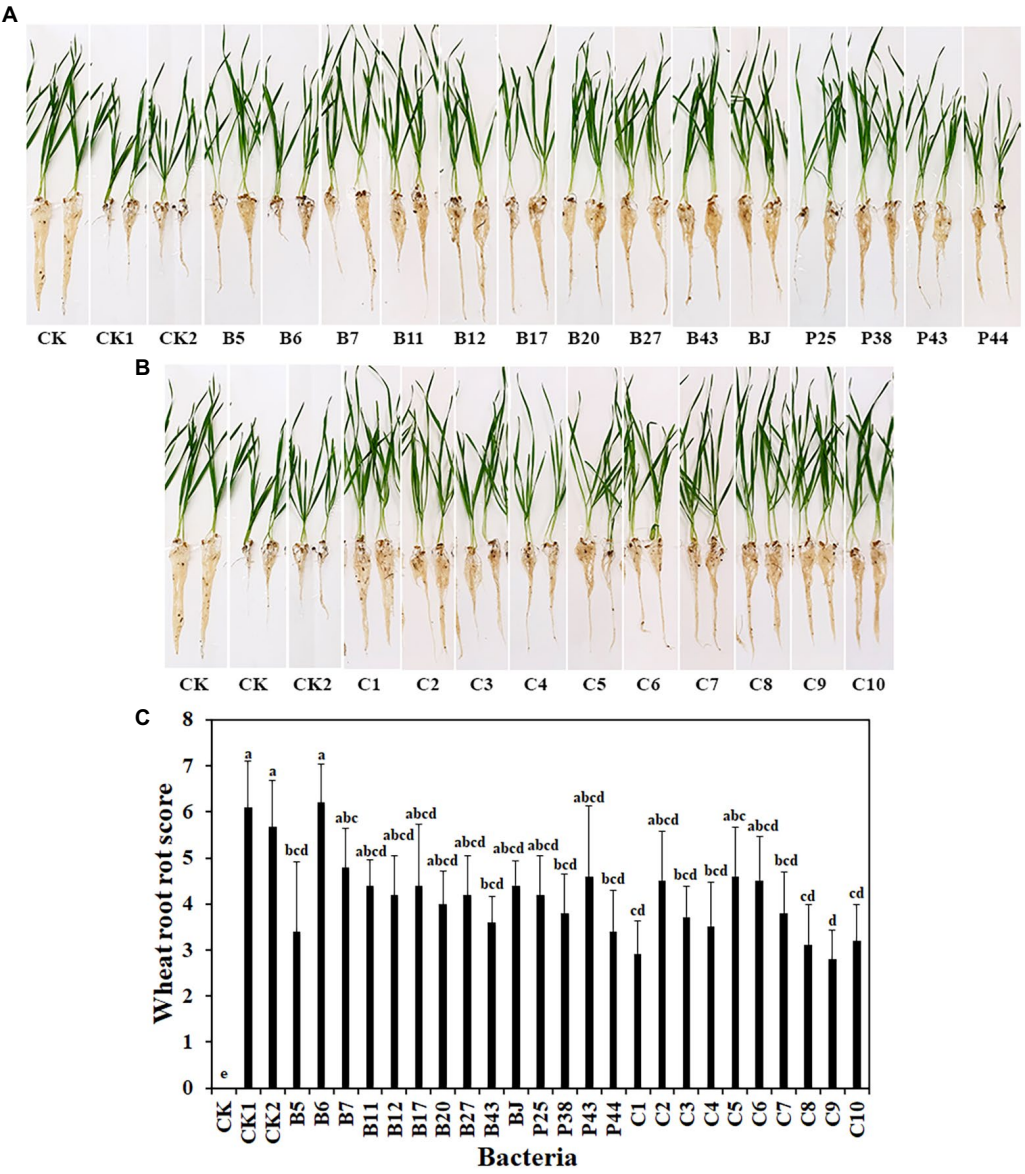
Effects of bacteria on wheat root rot caused by *R. solani* AG8. **(A)**. Single bacterial strain. **(B)**. SynComs. **(C)**. Wheat root rot scores. CK: Wheat grown in soil without bacteria and AG8 inoculation; CK1: AG8 only; CK2: AG8 and ddH_2_O; B5: AG8 and *Pseudomonas* sp. B5; B6: AG8 and *Streptomyces* sp. B6; B7: AG8 and *Chryseobacterium* sp. B7; B11: AG8 and *Pseudomonas* sp. B11; B12: AG8 and *Pseudomonas* sp. B12; B17: AG8 and *Sphingomonas* sp. B17; B20: AG8 and *Cupriavidus campinensis* B20; B27: AG8 and *Asticcacaulis* sp. B27; B43: AG8 and *Rhodococcus erythropolis* B43; BJ: AG8 and *Janthinobacterium lividum* BJ; P25: AG8 and *Pseudomonas* sp. P25; P38: AG8 and *Chryseobacterium soldanellicola* P38; P43: AG8 and *Chryseobacterium* sp. P43; P44: AG8 and *Pedobacter* sp. P44; C1: AG8 and SynCom 1; C2: AG8 and SynCom 2; C3: AG8 and SynCom 3; C4: AG8 and SynCom 4; C5: AG8 and SynCom 5; C6: AG8 and SynCom 6; C7: AG8 and SynCom 7; C8: AG8 and SynCom 8; C9: AG8 and SynCom 9; C10: AG8 and SynCom 10. The values are means ± SD. Different letters indicate significant differences (*p* ≤ 0.05, Tukey’s test, *n* = 10).

To address the limitations of *in vitro* experiments, the effects of bacteria, including 14 single strains and 10 SynComs, on wheat root rot disease caused by *R. solani* AG8 were evaluated in soil in the greenhouse. After 3 weeks, compared with wheat growth in AG8 uninoculated soil (CK), susceptible wheat cultivar Alpowa were stunted and displayed severe root rot diseases in AG8 infested soil, while wheat treated with some single bacterial strains and SynComs reduced root rot at different levels (Figures 2A,B). Among them, wheat treated with single bacterial strains (*Pseudomonas* sp. B5, *Rhodococcus erythropolis* B43, *Chryseobacterium soldanellicola* P38, and *Pedobacter* sp. P44) and SynComs (C1, C3, C4, C7, C8, C9, and C10) significantly reduced wheat root rot score, compared with the controls (CK1 and CK2) in AG8 inoculated soil (Figure 2C). Further, the fresh root weight of wheat treated with single bacterial strains (*Pseudomonas* spp. B5 and B11, and *Chryseobacterium* sp. B7) and SynComs (C1, C4, C7, C8, C9, and C10) were greater than those of the controls in AG8 inoculated soil (Supplementary Figure 1). Collectively, the results showed that most SynComs protected wheat from AG8 infection. Overall, SynComs did not perform better than single strains, but were not inferior to single stains either. However, seven out of 10 SynComs significantly reduced disease, compared to only four out of 14 single strains. Six out of 10 SynComs increased root fresh weight, compared to only three out of 14 single strains.

### Bacteria impacted the root growth of *Arabidopsis thaliana*

**Figure 3:**
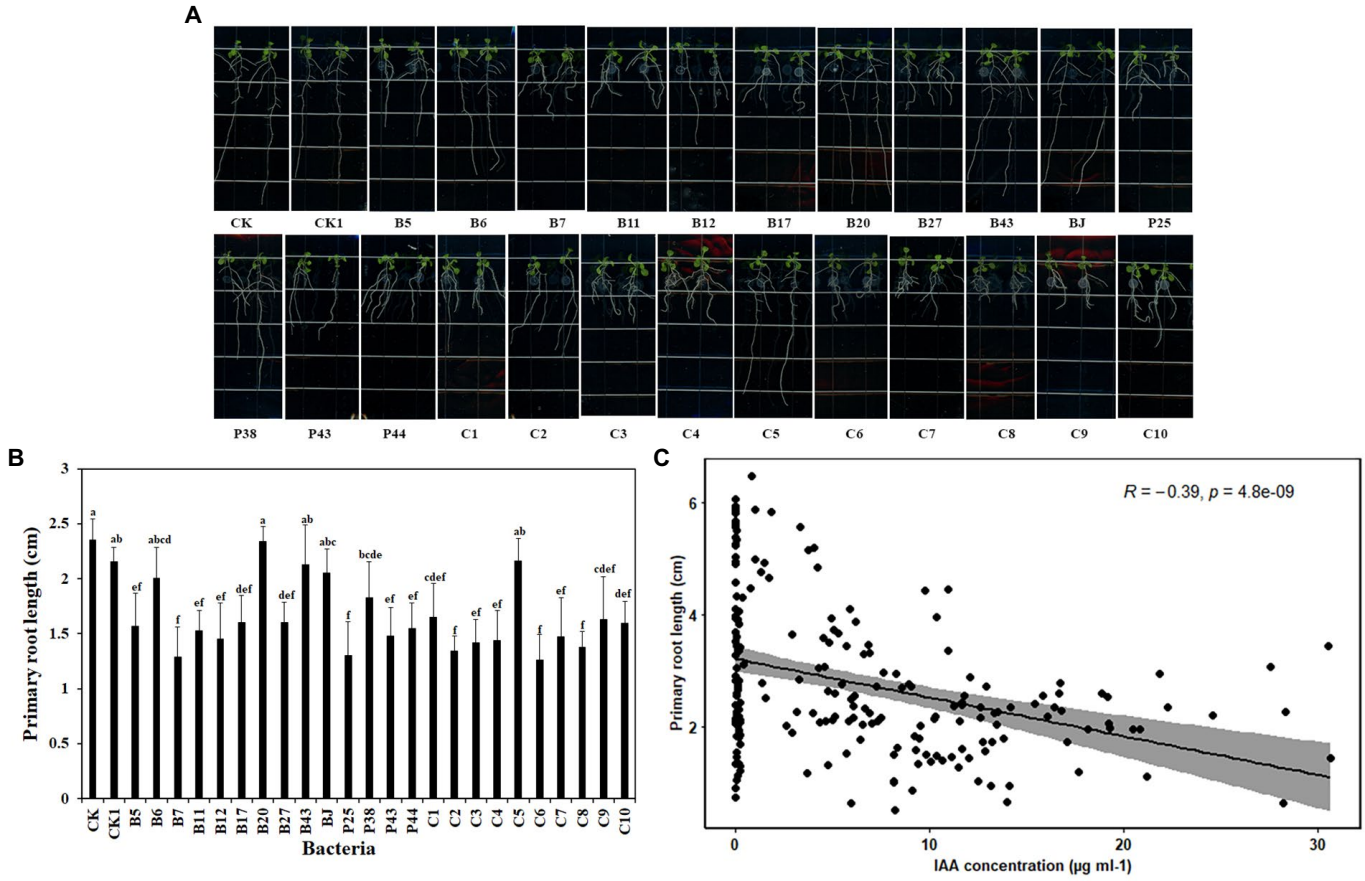
Effects of bacteria on root growth of *Arabidopsis thaliana*. **(A)**. The root system of *A. thaliana* grown on MS medium. **(B)**. The square-root-transformed primary root length of *Arabidopsis*. **(C)**. The correlation of the primary root length of *Arabidopsis* and IAA concentration. CK: *Arabidopsis* only; CK1: *Arabidopsis* and ddH_2_O; B5: *Arabidopsis* and *Pseudomonas* sp. B5; B6: *Arabidopsis* and *Streptomyces* sp. B6; B7: *Arabidopsis* and *Chryseobacterium* sp. B7; B11: *Arabidopsis* and *Pseudomonas* sp. B11; B12: *Arabidopsis* and *Pseudomonas* sp. B12; B17: *Arabidopsis* and *Sphingomonas* sp. B17; B20: *Arabidopsis* and *Cupriavidus campinensis* B20; B27: *Arabidopsis* and *Asticcacaulis* sp. B27; B43: *Arabidopsis* and *Rhodococcus erythropolis* B43; BJ: *Arabidopsis* and *Janthinobacterium lividum* BJ; P25: *Arabidopsis* and *Pseudomonas* sp. P25; P38: *Arabidopsis* and *Chryseobacterium soldanellicola* P38; P43: *Arabidopsis* and *Chryseobacterium* sp. P43; P44: *Arabidopsis* and *Pedobacter* sp. P44; C1: *Arabidopsis* and SynCom 1; C2: *Arabidopsis* and SynCom 2; C3: *Arabidopsis* and SynCom 3; C4: *Arabidopsis* and SynCom 4; C5: *Arabidopsis* and SynCom 5; C6: *Arabidopsis* and SynCom 6; C7: *Arabidopsis* and SynCom 7; C8: *Arabidopsis* and SynCom 8; C9: *Arabidopsis* and SynCom 9; C10: *Arabidopsis* and SynCom 10. The values are means ± SD. Different letters indicate significant differences (*p* ≤ 0.05, Tukey’s test, *n* = 9).

Beneficial microbes promote plant growth through various mechanisms. To explore the basis of the effects of the tested bacteria on plant growth, *Arabidopsis* was used because it has a short life cycle and is suitable to be grown *in vitro*. Six-day-old *Arabidopsis* seedlings were placed vertically on MS agar Petri dishes with 2 μl bacteria culture (OD_600_ = 0.1) at the end of the primary root. After 7 days of co-cultivation, nine of 14 bacteria significantly reduced the primary root length of *Arabidopsis* seedlings, compared with nontreated roots (Figures 3A,B). They were *Pseudomonas* sp. B5, *Chryseobacterium* sp. B7, *Pseudomonas* sp. B11, *Pseudomonas* sp. B12, *Sphingomonas* sp. B17, *Asticcacaulis* sp. B27, *Pseudomonas* sp. P25, *Chryseobacterium* sp. P43, and *Pedobacter* sp. P44. Similar reductions in the primary root length of *Arabidopsis* seedlings were observed in all SynComs except C5 (Figures 3A,B). Together, these data showed that bacteria impacted the root growth of *A. thaliana*.

Auxin has a critical role in plant root development (Dubrovsky et al., 2018). To investigate whether bacteria produce auxin, the total content of auxin was examined in the culture supernatant of bacteria. Interestingly, a large proportion of the tested bacteria, including 10 single strains and all 10 SynComs, produced auxin at various concentrations. The 10 single strains were *Pseudomonas* sp. B5, *Streptomyces* sp. B6, *Chryseobacterium* sp. B7, *Pseudomonas* sp. B11, *Pseudomonas* sp. B12, *Sphingomonas* sp. B17, *Asticcacaulis* sp. B27, *Chryseobacterium soldanellicola* P38, *Chryseobacterium* sp. P43, and *Pedobacter* sp. P44, in which the auxin concentration ranged from 0.05 ± 0.01 μg ml^−1^ to 24.60 ± 6.19 μg ml^−1^ (Table 3). Further, a correlation analysis showed that primary root length of *Arabidopsis* had a negative correlation with IAA concentration (*r*^2^ = −0.39, *p* = 4.8e-09; Figure 3C). These results indicated that IAA produced by soilborne beneficial bacteria may influence plant performance.

**Table 3:** Indole acetic acid (IAA) produced by bacteria.

Bacteria	IAA^a^	Bacteria	IAA
	(μg ml^−1^)		(μg ml^−1^)
*Pseudomonas* sp. B5	12.18 ± 1.48	*Chryseobacterium* sp. P43	0.05 ± 0.01
*Streptomyces* sp. B6	4.40 ± 0.80	*Pedobacter* sp. P44	0.12 ± 0.03
*Chryseobacterium* sp. B7	11.74 ± 1.90	SynCom 1(C1)	0.20 ± 0.03
*Pseudomonas* sp. B11	18.04 ± 2.74	SynCom 2(C2)	0.18 ± 0.04
*Pseudomonas* sp. B12	24.60 ± 6.19	SynCom 3(C3)	6.77 ± 2.78
*Sphingomonas* sp. B17	5.66 ± 0.73	SynCom 4(C4)	4.77 ± 1.71
*Cupriavidus campinensis* B20	0.00 ± 0.00	SynCom 5(C5)	0.89 ± 0.66
*Asticcacaulis* sp. B27	4.28 ± 1.42	SynCom 6(C6)	11.76 ± 2.83
*Rhodococcus erythropolis* B43	0.01 ± 0.01	SynCom 7(C7)	10.87 ± 2.54
*Janthinobacterium lividum* BJ	0.00 ± 0.00	SynCom 8(C8)	17.08 ± 3.35
*Pseudomonas* sp. P25	0.00 ± 0.00	SynCom 9(C9)	7.75 ± 3.30
*Chryseobacterium soldanellicola* P38	0.07 ± 0.01	SynCom 10(C10)	7.66 ± 1.10

a The values are means ± standard deviation (SD, n = 9).

### Volatiles of bacteria inhibited the growth of *Rhizoctonia solani* AG8

**Figure 4:**
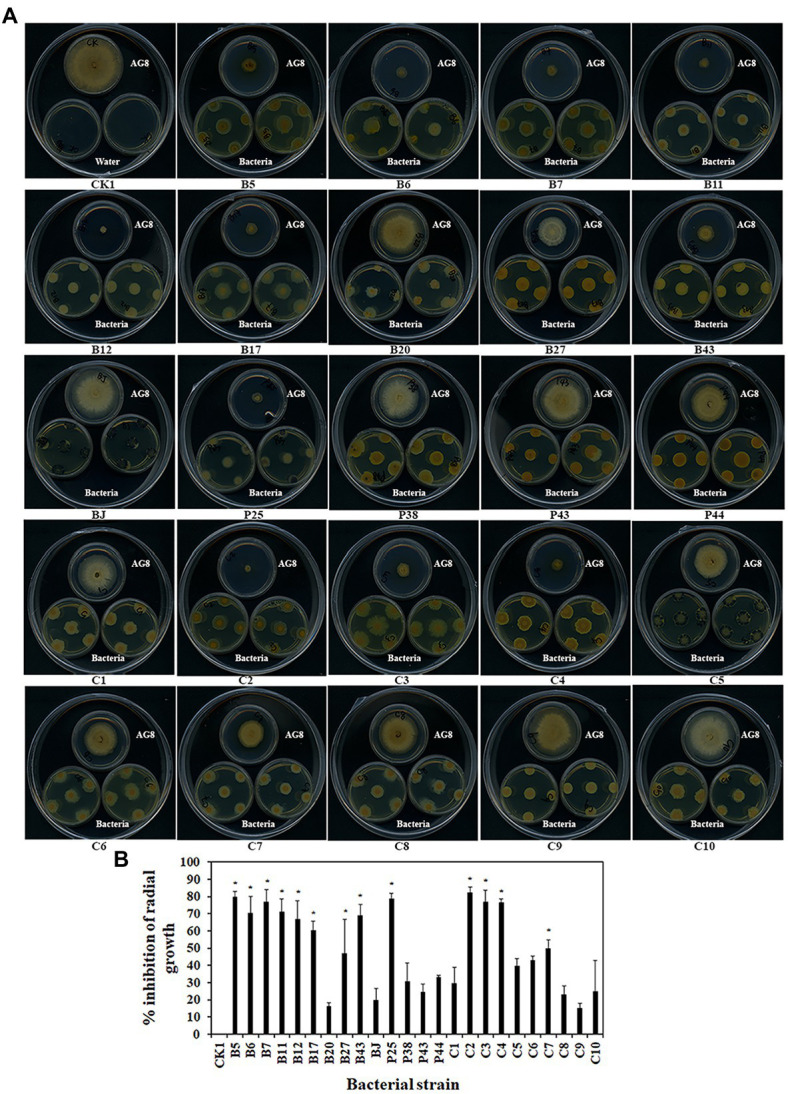
Effects of bacterial volatiles on the growth of *R. solani* AG8. **(A)**. The growth of AG8. **(B)**. Inhibition of radial growth of AG8. CK1: ddH_2_O; B5: *Pseudomonas* sp. B5; B6: *Streptomyces* sp. B6; B7: *Chryseobacterium* sp. B7; B11: *Pseudomonas* sp. B11; B12: *Pseudomonas* sp. B12; B17: *Sphingomonas* sp. B17; B20: *Cupriavidus campinensis* B20; B27: *Asticcacaulis* sp. B27; B43: *Rhodococcus erythropolis* B43; BJ: *Janthinobacterium lividum* BJ; P25: *Pseudomonas* sp. P25; P38: *Chryseobacterium soldanellicola* P38; P43: *Chryseobacterium* sp. P43; P44: *Pedobacter* sp. P44; C1: SynCom 1; C2: SynCom 2; C3: SynCom 3; C4: SynCom 4; C5: SynCom 5; C6: SynCom 6; C7: SynCom 7; C8: SynCom 8; C9: SynCom 9; C10: SynCom 10. The values are means ± SD. Asterisks indicate significant differences (*p* ≤ 0.05, Dunn test, *n* = 3).

Some bacteria may interact with fungi by producing volatiles. The effects of bacterial volatiles on the growth of AG8 were investigated using a large Petri dish containing three small Petri dishes. Interestingly, the volatiles emitted by nine single strains and four SynComs significantly inhibited the growth of AG8 (Figures 4A,B). They included four *Pseudomonas* spp. B5, B11, B12, and P25, *Streptomyces* sp. B6*, Chryseobacterium* sp. B7, *Sphingomonas* sp. B17, *Asticcacaulis* sp. B27, *Rhodococcus erythropolis* B43, and SynComs C2-4, C7. The inhibition ranged from 49.76 ± 4.95 to 82.60% ± 2.95% (Figure 4B).

### Antifungal activity of cell-free supernatants

**Figure 5:**
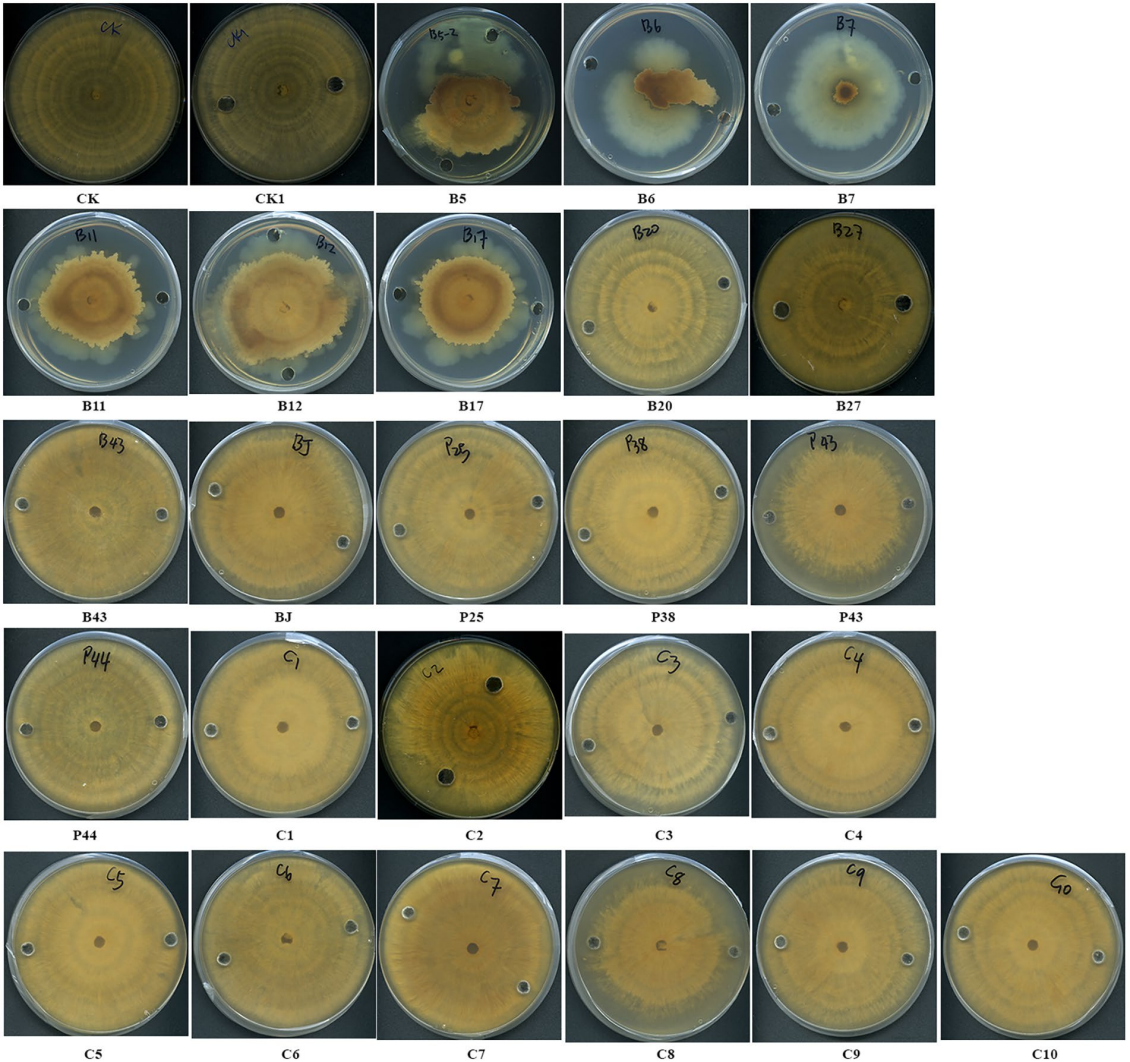
Effects of cell-free supernatants (CFSs) of bacteria on the growth of *R. solani* AG8. CK: AG8 only; CK1: ddH_2_O; B5: CFSs of *Pseudomonas* sp. B5; B6: CFSs of *Streptomyces* sp. B6; B7: CFSs of *Chryseobacterium* sp. B7; B11: CFSs of *Pseudomonas* sp. B11; B12: CFSs of *Pseudomonas* sp. B12; B17: CFSs of *Sphingomonas* sp. B17; B20: CFSs of *Cupriavidus campinensis* B20; B27: CFSs of *Asticcacaulis* sp. B27; B43: CFSs of *Rhodococcus erythropolis* B43; BJ: CFSs of *Janthinobacterium lividum* BJ; P25: CFSs of *Pseudomonas* sp. P25; P38: CFSs of *Chryseobacterium soldanellicola* P38; P43: CFSs of *Chryseobacterium* sp. P43; P44: CFSs of *Pedobacter* sp. P44; C1: CFSs of SynCom 1; C2: CFSs of SynCom 2; C3: CFSs of SynCom 3; C4: CFSs of SynCom 4; C5: CFSs of SynCom 5; C6: CFSs of SynCom 6; C7: CFSs of SynCom 7; C8: CFSs of SynCom 8; C9: CFSs of SynCom 9; C10: CFSs of SynCom 10.

The antifungal activities of cell-free supernatants of bacteria were examined. After 1 week of incubation, the margins of fungal cultures treated with the culture supernatants of three *Pseudomonas* sp. strains (B5, B11, and B12), *Streptomyces* sp. B6, *Chryseobacterium* sp. B7, and *Sphingomonas* sp. B17 became irregular shaped and creamy colored (Figure 5). The growth of AG8 treated with these bacterial strains was significantly inhibited and the inhibition ranged from 30.50 ± 8.99 to 76.56% ± 3.60%, compared with the controls (Table 4). However, the cell-free supernatants from the rest of the eight bacteria and 10 SynComs did not show any antifungal activities.

**Table 4:** Inhibition of cell-free supernatants of bacteria on the radial growth of *R. solani* AG8.

Bacteria	% Inhibition of radial growth^a^
Control (*R. solani* AG8 only)	0
Control (ddH_2_O)	0
*Pseudomonas* sp. B5	40.62 ± 9.10^*^
*Streptomyces* sp. B6	57.53 ± 15.53^*^
*Chryseobacterium* sp. B7	76.56 ± 3.60^*^
*Pseudomonas* sp. B11	48.45 ± 18.90^*^
*Pseudomonas* sp. B12	30.50 ± 8.99^*^
*Sphingomonas* sp. B17	42.41 ± 1.51^*^

a The values are means ± standard deviation (SD, n = 3).

* Indicate significant differences (p ≤ 0.05, Dunn test).

### Ability of bacteria to produce hydrogen cyanide

**Figure 6:**
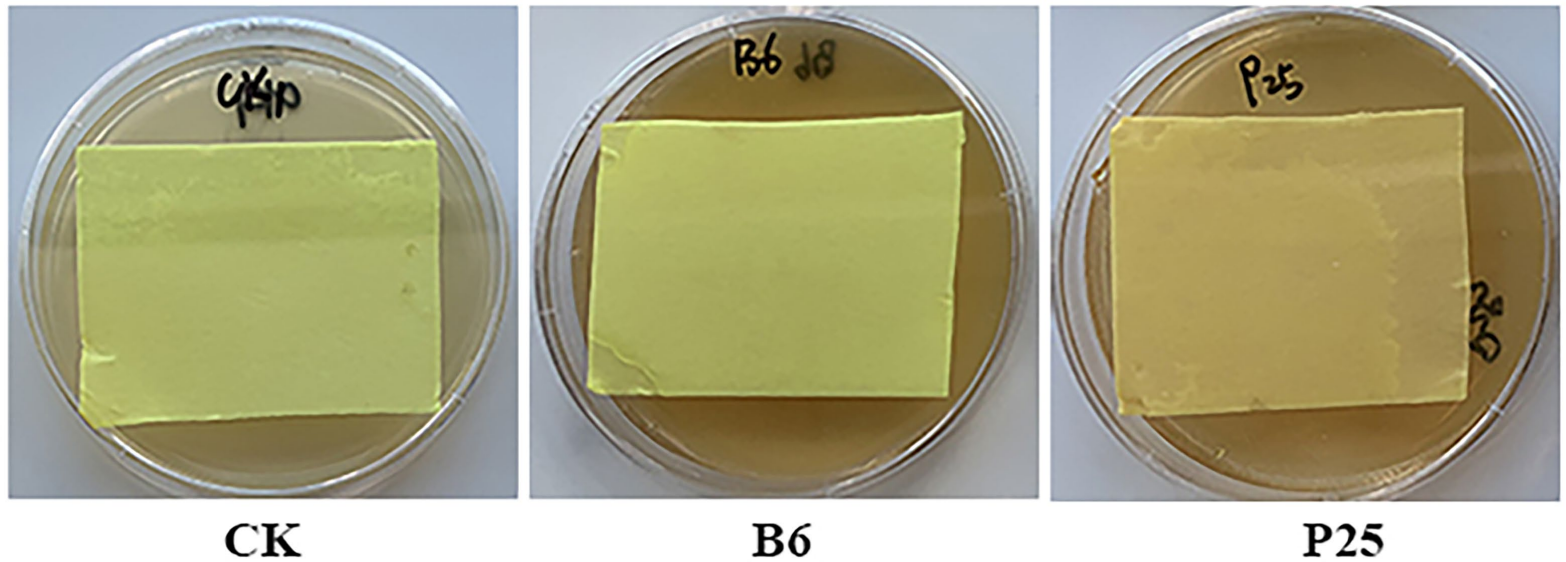
Hydrocyanic acid (HCN) produced by bacteria. CK: without bacteria; B6: *Streptomyces* sp. B6; P25: *Pseudomonas* sp. P25.

Some bacteria can produce hydrogen cyanide, an inorganic compound, that showed antagonistic activity against pathogens (Voisard et al., 1989; Hyder et al., 2020). Of the tested bacteria in this study, the filter paper located in the lid of the petri dish culturing *Pseudomonas* sp. P25 displayed light brown color, indicating only *Pseudomonas* sp. P25 was able to produce weak HCN (Figure 6; Supplementary Figure 2).

## Discussion

Microbial communities have been widely recognized to perform various functions that are beneficial in agriculture systems. In recent years, synthetic microbial communities (SynComs) offer a unique opportunity to improve crop production through enhancing the naturally existing community functions (Großkopf and Soyer, 2014; Eng and Borenstein, 2019; de Souza et al., 2020). In our previous studies, a collection of bacteria was isolated from wheat rhizosphere and displayed antifungal activity against soilborne fungal pathogens *in vitro* and/or *in vivo* (Yin et al., 2013, 2021). Here we extended this work, created 10 SynComs with different compositions from 14 bacteria, and seven of them protected wheat from *Rhizoctonia solani* AG8 infection. Consistent with previous reports (Bakker et al., 2007; Glick, 2012; Abd El-Rahman et al., 2019; Alemneh et al., 2020), the bacteria tested in this study interact with fungi and plants through various mechanisms, including producing auxin, volatiles, HCN, and secreted compounds into cultures. Together, our results indicated that the interaction of microbes and plant is complex. The creation of SynComs using lab-selected bacteria may provide a potential to improve plant performance and may have more resilience and stability compared to single strains, which are supported by the findings of Minchev et al. (2021). However, further study is needed for practical application in agriculture.

The absence of resistant wheat varieties and few management strategies for controlling *Rhizoctonia* root rot diseases (Smith et al., 2003; Schroeder and Paulitz, 2008; Okubara and Jones, 2011) require development of effective strategies to manage the disease. Natural suppression of *Rhizoctonia* in long-term wheat plantings (Roget et al., 1999; Yin et al., 2013; Schillinger and Paulitz, 2014) and the roles of soil microbial communities in this process (Yin et al., 2013; Donn et al., 2014) open a door to use microbes to manage this disease. The microbial communities from wheat or other crop rhizospheres were well characterized in numerous studies using high-throughput sequencing technologies and some beneficial microbes were enriched in plant rhizosphere and endophytic roots (Carrión et al., 2019; Tsolakidou et al., 2019; Yin et al., 2021). Using cultivation-based approaches, 14 bacteria were identified from the wheat rhizosphere belonging to nine families that have antifungal activities against AG8 *in vitro* and some bacteria reduced wheat root rot disease (Yin et al., 2013, 2021). Among 14 tested bacteria, four bacteria belong to genera *Pseudomonas* and three bacteria were classified to genera *Chryseobacterium. Pseudomonas* spp. and *Chryseobacterium* spp. are ubiquitous bacteria in agricultural soils and are among the most abundant genera in wheat rhizosphere (Yin et al., 2013; Schlatter et al., 2020; Kavamura et al., 2021). Many soilborne *Pseudomonas* strains suppress plant pathogens and promote plant growth and development (Weller, 2007; Berg et al., 2017). The results of IAA, cell-free component and HCN implied that *Pseudomonas* sp. 25 is apparently different from *Pseudomonas* sp. B5, B11, and B12. Several *Chryseobacterium* species, such as *C. soldanella*, *C. endophyticum*, *C. indologenes,* and *C. balustinum* were reported to protect plants from pathogen infection (Solano et al., 2008; Yin et al., 2013; Sang et al., 2018). Interestingly, *Chryseobacterium soldanellicola* not only suppresses AG8 infection but protects plants from bacterial wilt and Phytophthora blight (Yoo et al., 2020), and promotes plant growth (Yoo et al., 2020). The multifunction features and high abundance in the agricultural soil (Yin et al., 2013, 2021) indicate the importance of *C. soldanellicola* in the wheat rhizosphere. In addition, *Rhodococcus erythropolis* B43 reduced wheat root rot in this study. *Rhodococcus erythropolis* has been proposed as a biocontrol agent for plant protection against soft-rot bacteria (Barbey et al., 2013). To our knowledge, this is the first report that *R. erythropolis* is able to protect wheat from AG8 infection. Although *Streptomyces* sp. B6 could inhibit the growth of AG8 *in vitro* dual-culture assay (Yin et al., 2021), treatment of susceptible wheat seeds with *Streptomyces* sp. B6 did not suppress the incidence of root rot disease in AG8 inoculated soil, so severe stunting of wheat was observed. In contrast, C9 displayed an opposite trend, suggesting that the phenotypic output of *in vitro* assays is not always consistent with that of an *in vivo* test. It might be because bacteria interact with existing microbes in the soil which may influence the antagonistic activities or reduce their stability resulting in low survival in the soil. A similar phenomenon occurred for *Pseudomonas* sp. (Yin et al., 2013) and *in vitro* test with *Arabidopsis* for *Bacillus* strains did not support the results on tomato plants in pots (Tsolakidou et al., 2019). Therefore, testing in soil and crop fields is key for practical applications. Comprehensive culture collections of beneficial bacteria not only provide a potentially highly valuable resource for crop productivity, especially under adverse environmental and biotic stresses, but are a prerequisite for creating consortia of microbes. So far, microbe collections are largely focused on bacteria and more efforts need to be extended to other microbial categories such as fungi and oomycetes.

It is unlikely that a single beneficial microbe inoculum will provide an efficient function because of the complexity and diversity of natural microbial ecosystems. This study focused on bacterial consortia rather than single strains. The selection of the optimal microbial species is critical to building an effective microbial consortium. Many factors can be considered, such as microorganism’s growth features, nutritional requirements, hormones, and secreted organic or chemical compounds. Abundant studies developed various models to design SynComs (Paredes et al., 2018; Eng and Borenstein, 2019; McCarty and Ledesma-Amaro, 2019; Karkaria et al., 2021). The rationale for the construction of SynComs in this study was to combine the same genera species, select stronger antifungal levels, and reduce the consortia complexity. SynCom C1, composed of four different *Pseudomonas* members, significantly reduced wheat root rot and increased root weight, compared with the controls, although only one of the separate components reduced disease (B5). Zhuang et al. (2021) reported that a synthetic community with six *Pseudomonas* strains promoted radish seedling growth. However, C2 composed of three *Chryseobacterium* members did not protect wheat from AG8. SynComs consisting of different bacterial genera also showed the ability to reduce disease, such as C8, C9, and C10, in this study. There are other examples of mixing genera in the literature. Co-inoculated *Pseudomonas* and *Burkholderia* bacterial strains increased *Arabidopsis* root area (Timm et al., 2016). Tsolakidou et al. (2019) found that SynComs consisting of 25 different bacterial genera promoted tomato growth. Interestingly, *Pseudomonas* sp. B12 exists in four SynComs (C1, C8, C9, and C10) with the most effective disease reductions (Table 1). When only *Pseudomonas* sp. B12 was replaced by *Rhodococcus erythropolis* B43, the effect of C3 on root rot marginally reduced, compared with C8. These data suggested that *Pseudomonas* sp. B12 appears to be important in these SynComs. In another study (Niu et al., 2017), a synthetic bacterial community consisting of seven strains was designed that inhibited the phytopathogenic fungus *Fusarium verticillioides* and the removal of one strain, *Enterobacter cloacae*, caused dramatic changes in the community compositions. These findings supported that removing one strain at a time is an effective approach to probing the role of each microbe in SynCom design (de Souza et al., 2020; Krumbach et al., 2021).

Creation or construction of SynComs is generally expected to enhance the abilities or functions of single strains. Some studies have reported that the created SynComs or mixtures produced synergistic effects (Sylla et al., 2015; Berendsen et al., 2018; Tsolakidou et al., 2019; Chaves-Gómez et al., 2021; Yang et al., 2021). In particular, Fujiwara et al. (2016) showed that individual stains were not effective against *Fusarium oxysporum* alone but were in combination. However, the synergistic or additive effects do not always occur. Consortia showed similar or even reduced (negative effects) abilities compared to the individuals (Freeman et al., 2004; Xu et al., 2010; Minchev et al., 2021). Further, Xu et al. (2011) showed that antagonistic interactions were more common, and synergistic effects were rare. In our study, seven SynComs significantly reduced wheat root rot disease, compared to the controls. None of them was significantly better than a single strain, but similar to the best-performing individual within the SynComs and no negative interactions were observed. This fits the model of no interaction. Similar phenomena were reported in the recent work (Minchev et al., 2021) that the microbial consortia effectively reduced tomato root and foliar diseases but were not more effective than the best single strains. Additionally, in our study, a greater percentage of the SynComs were effective than the single strains, but this could be due to the increased chances of getting effective strains when they were combined into mixtures.

SynCom stability is another desired feature for future applications. We grew the individual members of SynComs separately and then mixed them with the same concentration to prevent the extinction of one or more species and improve the functional stability of the bacteria consortium in short term. While not assessed here, investigation of the fate of those inoculations in the soil after plant growth is needed to address their persistence and stability; this will be required to fully exploit SynComs for future applications in agricultural ecosystems. To date, much of the exploration of synthetic microbial communities has been limited to the lab stages, but some biotechnological companies are working on microbial consortia for biostimulant products. For example, BioConsortia from California, United States^1^ developed a number of biostimulant products that are in the registration phase. Agronutrition from France is working on microbial consortia for biofertilizer to improve soil functions.^2^

Understanding the mechanisms underlying the interaction between microbes and plant hosts is particularly important to harness the power of microbiomes. Beneficial microbes stimulate plant growth through various mechanisms including direct and indirect effects (Persello-Cartieaux et al., 2003; Ryu et al., 2005; Bakker et al., 2007; Mazurier et al., 2009; Glick, 2012; Abd El-Rahman et al., 2019). Auxin is a key regulator of plant root growth and development (Overvoorde et al., 2010; Dubrovsky et al., 2018). Numerous studies reported that an increase in auxin concentration inhibited root elongation and promoted the development of lateral roots in a dose-dependent matter (Peret et al., 2009; Overvoorde et al., 2010). Auxin may also be involved in plant and microbe interactions. Specifically, microbes change plant root architecture through auxin or auxin-signaling pathways (Sukumar et al., 2013; Zamioudis et al., 2013; Matthiadis et al., 2020). Not surprisingly, our study found that 10 individuals and 10 SynComs could produce different amounts of auxin. Most of them reduced the primary root length of *Arabidopsis* (Figure 3B). Further correlation analysis showed the primary root length was negatively correlated with IAA concentration (Figure 3C). However, there were a few exceptions. *Streptomyces* sp. B6 could produce auxin but did not reduce the primary root length. In contrast, *Pseudomonas* sp. P25 showed the opposite result, indicating different mechanisms likely to occur. IAA levels produced by C1 were much lower than the three individual *Pseudomonas* sp. B5, B11, and B12 (Table 3). The inhibition of AG8 growth by C1 volatiles was smaller than the individual *Pseudomonas* as well. These data suggested that the synergistic effects of C1 consortia were not *via* IAA and volatiles. Volatiles present in natural soil contribute to fungal pathogen inhibition and a few studies have shown that volatiles emitted from cultivable bacteria had negative effects on fungal growth (Garbeva et al., 2011; van Agtmaal et al., 2018; de Boer et al., 2019; Li et al., 2020). Consistent with those studies, we found that the volatiles emitted by nine individuals and four SynComs significantly inhibited the growth of AG8. To date, more than 1,000 bacterial volatile compounds have been described including organic and inorganic compounds (Kai et al., 2009; Lemfack et al., 2014; Audrain et al., 2015; Hol et al., 2015; Li et al., 2020). The components of volatiles emitted by the tested bacteria require further investigation. Small inorganic volatiles like hydrogen cyanide have been recognized to be toxic to fungal pathogens and current reported bacteria include some species of *Pseudomonas*, *Chromobacterium*, and *Rhizobium* (Audrain et al., 2015; Piechulla et al., 2017; Abd El-Rahman et al., 2019; Hyder et al., 2020). In this study, only one *Pseudomonas* sp. P25 was able to produce hydrogen cyanide. However, Rijavec and Lapanje (2016) disproved the biocontrol function of HCN: they found no correlation between the level of HCN derived from the rhizobacteria and biocontrol effects. But they found that HCN indirectly increases the availability of phosphate. Thus, it was too early to conclude the antifungal activity of *Pseudomonas* sp. P25 is related to HCN. In addition, the cell-free supernatants of three *Pseudomonas* sp. B5, B11, and B12, *Streptomyces* sp. B6, *Chryseobacterium* sp. B7, and *Sphingomonas* sp. B17 appeared to degrade the margins of fungal cultures and inhibit the growth of AG8 (Figure 5; Table 4). The fungal cell wall consists of 22–44% chitin, which provides strength and toughness for fungal cell walls (Manjula and Podile, 2005; Gow et al., 2017). Biocontrol bacterial genera, including *Pseudomonas*, *Streptomyces*, and *Bacillus,* are known to produce cell wall degrading enzymes (Manjula and Podile, 2005; Schönbichler et al., 2020), which is a possible reason for the creamy irregular shape surrounding the fungal hyphae. To our knowledge, the antifungal activity of cell-free supernatant from *Sphingomonas* has not been reported before. We failed to detect antifungal activity of cell-free supernatants from earlier growth stage of bacteria (24-h bacterial growth, data not shown) suggesting bacteria produced and accumulated metabolites with protective effects in the late growth stage. Taken together, our exploration supports the notion that microbes utilize different mechanisms to interact with each other and plant hosts. However, a systematic investigation of different functional microbial members is needed to further reveal the functions and mechanisms of the root microbiome in plant growth and health.

## Conclusion

Our study shows that the seven SynComs derived from wheat rhizosphere protected wheat from *Rhizoctonia solani* AG8 infection, although none was significantly better than a single strain. No negative interactions were observed, meaning that SynComs were more likely to be effective than single strains. The isolated bacteria with beneficial features inhibited the growth of fungal pathogen and influenced plant properties *via* various mechanisms. SynCom is an emerging technique and alternative strategy through co-culturing multiple microbes to enhance the function of a microbiome to improve crop production.

## Data availability statement

The datasets presented in this study can be found in online repositories. The names of the repository/repositories and accession number(s) can be found in the article/Supplementary material.

## Author contributions

CY, CH, and TP conceived and designed the research and wrote the manuscript. CY performed the research and data analysis. All authors contributed to the article and approved the submitted version.

## Funding

This study was funded by the Oregon Wheat Commission, Washington Grain Commission, and USDA-ARS.

## Conflict of interest

The authors declare that the research was conducted in the absence of any commercial or financial relationships that could be construed as a potential conflict of interest.

## Publisher’s note

All claims expressed in this article are solely those of the authors and do not necessarily represent those of their affiliated organizations, or those of the publisher, the editors and the reviewers. Any product that may be evaluated in this article, or claim that may be made by its manufacturer, is not guaranteed or endorsed by the publisher.

## Footnotes

^1^ https://bioconsortia.com/

^2^ https://www.agronutrition.com/fr
