# Supplementary figures and images for: Synthetic microbial consortia derived from rhizosphere soil protect wheat against a soilborne fungal pathogen
Source: Front Microbiol. 2022 Aug 31;13:908981. doi: 10.3389/fmicb.2022.908981 (PMC9473337; doi:10.3389/fmicb.2022.908981)

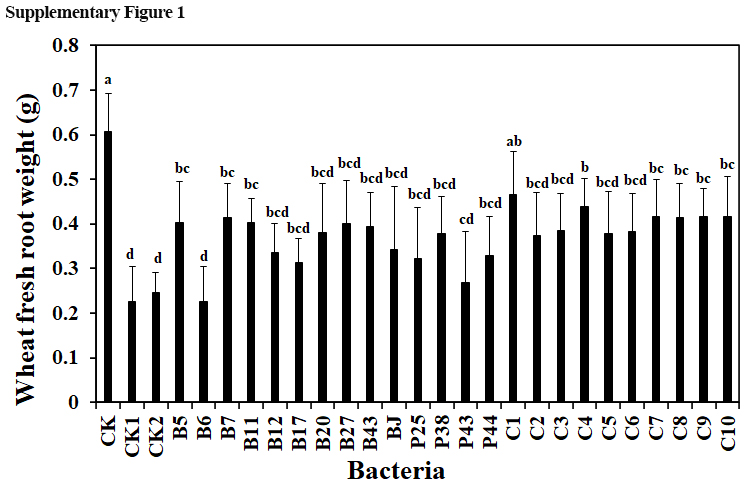

Supplement: Supplementary file 1 [file Image_1.JPEG]

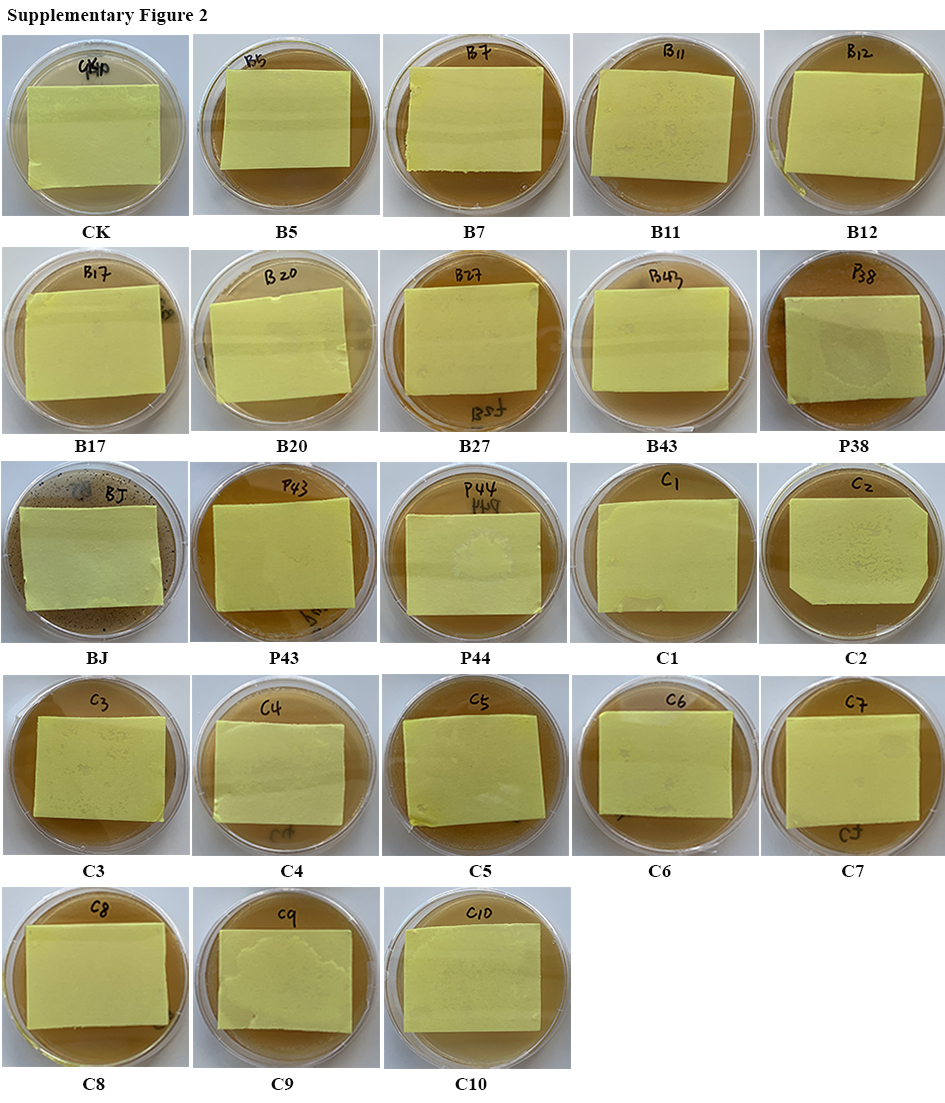

Supplement: Supplementary file 2 [file Image_2.JPEG]
